# Where Robotic Surgery Meets the Metaverse

**DOI:** 10.3390/cancers14246161

**Published:** 2022-12-14

**Authors:** Fijs W. B. van Leeuwen, Jos A. van der Hage

**Affiliations:** 1 Interventional Molecular Imaging Laboratory, Department of Radiology, Leiden University Medical Center, 2300 RC Leiden, The Netherlands; 2 Department of Sugery, Leiden University Medical Center, 2300 RC Leiden, The Netherlands

With a focus on hepatobiliary surgery, the review by Giannone et al. [1] touches upon the combination of two essential innovations in the field of minimally invasive robotic surgery: augmented reality (or rather, mixed-reality (MR) visualizations), and artificial intelligence (AI, which includes machine learning). The combination of these approaches is referred to by some as the surgical metaverse. This forward-thinking concept is gaining attention due to its promise to address the practical demand of surgeons for easier, more accurate, and, ultimately, safer interventions.

Instrumental for implementing these advanced technologies is the availability of a ‘control tower’ that facilitates the display and processing of multiple digital data streams [2], a role that suits the robotic console perfectly (Scheme 1). Hence, building on the wide surgical robot installation base could help create a global ecosystem that supports both innovation and implementation. While the authors limit their discussion to hepatobiliary applications, which form an up-and-coming field within robotic surgery, the addressed technologies are ubiquitous. In fact, these technologies have seen prior use in daily life, as well as in other robotic surgery applications [3]. This underlines the fact that universal principles of creativity form the basis of innovation. Additionally, following this line of reasoning, the best way to realize progress in the field of hepatobiliary surgery is to monitor related activities that occur across the surgical landscape.

During robotic surgery, image guidance is used to compensate for the lack of tactile feedback. As stated by the authors, the surgical demand herein can best be addressed by the integrated use of a number of complementary guidance strategies: (1) high-fidelity procedural planning based on preoperative 3D imaging roadmaps [4], (2) the superimposition of live data and 3D imagery on endoscopic views (e.g., MR visualizations and GPS-like navigation strategies; [5]), (3) dynamic lesion/tissue characterization via intraoperative imaging (e.g., drop-in ultrasound for in depth detection; [6]) or fluorescence imaging (superficial detection; [7]), and (4) the use of machine learning strategies to alleviate the mounting cognitive input, to advance the interpretation of the surgical data output, and to guide image-to-patient registration [2].

The most specific topic—and challenge—addressed by Giannone et al. [1] is the augmentation of endoscopic videos via the 3D rendering of preoperative imaging roadmaps, a feature that yields futuristic graphics, but which is also technically challenging and time-consuming. In rigid surgical applications, e.g., neuro-, maxillofacial-, and orthopedic-surgery, the use of navigation concepts based on similar technologies is evolving rapidly [8]. The major Achilles’ heel for the implementation of these concepts during soft-tissue surgery is tissue movement. Patient positioning and respiratory motion alone can cause superimposition inaccuracies at the scale of centimeters [9]. CO_2_ insufflation (commonly applied in abdominal robotic surgery) and surgical tissue manipulation inherently cause additional deformations that render any traditional landmark-based registration algorithm useless. The greatest possibility for the improvement of the registration accuracy lies in the real-time correction of movement artifacts. Meaning that registration should be “elastic”, thus requiring advanced deformable reconstruction algorithms that make use of active organ tracking, possibly strengthened by intraoperative imaging [10]. If the necessary computing power can be realized, state-of-the-art machine learning algorithms have the potential to guide the automated real-time “warping” of the renderings to match the “elasticity” of the tissue [11]. To reach this point, however, multispecialty consensus on surgical approaches is required [11], as well as the standardization of the technologies used during interventions, and coordinated large-scale data collection.

With the introduction of the new European medical device regulations (MDR), it has become increasingly complex to bring new imaging hard- and software into the surgical theatre. A feature that also impacts the surgical metaverse. Compliance with the more stringent regulations increases the development and CE-marking costs and ultimately enhances the probability of late clinical failure for prototype concepts. While these regulations limit the speed of translation and the number of technologies that will find their way into the clinic, the fact that only the sturdiest technologies will “survive” could potentially also prove to be beneficial. To anticipate this shift, the surgical scientific community will need to move away from the now widely adapted measure of success based on first-in-patient demonstration and devise innovative means to objectively relate technological innovations for the benefit of healthcare professionals and/or patients. To yield so-called performance-guided surgery, image-guided surgery technologies need to enhance surgical proficiency, improve clinical outcome, and/or provide a societal impact. This will be especially challenging to achieve for up-and-coming technologies that have not yet seen widespread clinical implementation. The best way to assess the potential of technologies requires assessment early in the research and development pipeline, for example, studying their ability to alter user actions. A concept that has been successfully used to determine the difference in the use of medical technologies between novices and experts e.g. during robotic surgery [12] and liver punctions [13]. The findings obtained during these assessments can be further corroborated by relating them to complication rates and outcome data [14].

The next logical step is to employ the metaverse in a training setting. This is especially relevant since the term surgical metaverse is also often used to describe a virtual training environment that closely mimics all activities associated with surgical procedures, i.e., target identification, assessment task completion, quality control, etc. In this setting, technologies such as mixed-reality visualizations—as the authors suggest—are particularly appealing during training for straightforward procedural actions such as port placement. Applications where the guidance provided can be macroscopic in nature. The standardization of proficiency scoring provides a challenge for such training programs. A clear example is the need to score the ability of new concepts to increase their utility [15]. The implementation of the metaverse in training can, however, be taken much further. For instance, the gamification of complete surgical procedures can be used to support and monitor the step-by-step transition from novice to expert, for both surgeons and complete surgical teams. Furthermore, mixed-reality displays can help support the future implementation of concepts such as telementoring.

In conclusion, while not yet matured for hepatobiliary surgery, the presented use of the surgical metaverse holds future potential. This potential can be capitalized on when the knowledge and expertise gained by different surgical disciplines are combined. That said, major scientific advances in the areas of image registration/interpretation and performance scoring are still required to ensure the more widespread implementation of image-to-patient registration during soft-tissue surgery.

## Figures and Tables

**Scheme 1:**
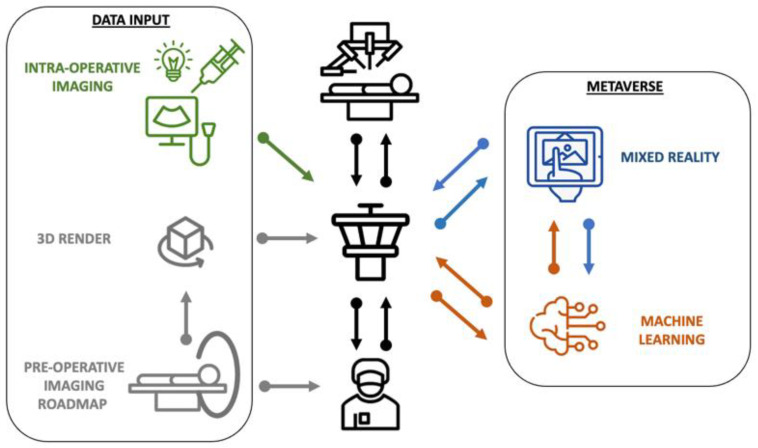
The relationship between pre- and intra-operative imaging within the robotic–surgical metaverse.

## References

[1] Giannone F., Felli E., Cherkaoui Z., Mascagni P., Pessaux P. (2021). Augmented Reality and Image-Guided Robotic Liver Surgery. Cancers.

[2] Wendler T., van Leeuwen F.W.B., Navab N., van Oosterom M.N. (2021). How molecular imaging will enable robotic precision surgery: The role of artificial intelligence, augmented reality, and navigation. Eur. J. Nucl. Med. Mol. Imaging.

[3] Greco F., Cadeddu J.A., Gill I.S., Kaouk J.H., Remzi M., Thompson R.H., van Leeuwen F.W.B., van der Poel H.G., Fornara P., Rassweiler J. (2014). Current Perspectives in the Use of Molecular Imaging To Target Surgical Treatments for Genitourinary Cancers. Eur. Urol..

[4] Alvarez Paez A.M., Brouwer O.R., Veenstra H.J., van der Hage J.A., Wouters M., Nieweg O.E., Valdés-Olmos R.A. (2012). Decisive role of SPECT/CT in localization of unusual periscapular sentinel nodes in patients with posterior trunk melanoma: Three illustrative cases and a review of the literature. Melanoma Res..

[5] Porpiglia F., Checcucci E., Amparore D., Piramide F., Volpi G., Granato S., Verri P., Manfredi M., Bellin A., Piazzolla P. (2020). Three-dimensional Augmented Reality Robot-assisted Partial Nephrectomy in Case of Complex Tumours (PADUA ≥ 10): A New Intraoperative Tool Overcoming the Ultrasound Guidance. Eur. Urol..

[6] Schneider C., Nguan C., Rohling R., Salcudean S. (2016). Tracked “Pick-Up” Ultrasound for Robot-Assisted Minimally Invasive Surgery. IEEE Trans. Biomed. Eng..

[7] van Oosterom M.N., van der Poel H.G., Navab N., van de Velde C.J.H., van Leeuwen F.W.B. (2018). Computer-assisted surgery: Virtual- and augmented-reality displays for navigation during urological interventions. Curr. Opin. Urol..

[8] Mezger U., Jendrewski C., Bartels M. (2013). Navigation in surgery. Langenbecks Arch. Surg..

[9] Meershoek P., van den Berg N.S., Lutjeboer J., Burgmans M.C., van der Meer R.W., van Rijswijk C.S.P., van Oosterom M.N., van Erkel A.R., van Leeuwen F.W.B. (2021). Assessing the value of volume navigation during ultrasound-guided radiofrequency- and microwave-ablations of liver lesions. Eur. J. Radiol. Open.

[10] Amparore D., Checcucci E., Piazzolla P., Piramide F., De Cillis S., Piana A., Verri P., Manfredi M., Fiori C., Vezzetti E. (2022). Indocyanine Green Drives Computer Vision Based 3D Augmented Reality Robot Assisted Partial Nephrectomy: The Beginning of “Automatic” Overlapping Era. Urology.

[11] Ma R., Vanstrum E.B., Lee R., Chen J., Hung A.J. (2020). Machine learning in the optimization of robotics in the operative field. Curr. Opin. Urol..

[12] Judkins T.N., Oleynikov D., Stergiou N. (2009). Objective evaluation of expert and novice performance during robotic surgical training tasks. Surg. Endosc..

[13] Boekestijn I., Azargoshasb S., van Oosterom M.N., Slof L.J., Dibbets-Schneider P., Dankelman J., van Erkel A.R., Rietbergen D.D.D., van Leeuwen F.W.B. (2022). Value-assessment of computer-assisted navigation strategies during percutaneous needle placement. Int. J. Comput. Assist. Radiol. Surg..

[14] Hung A.J., Chen J., Che Z., Nilanon T., Jarc A., Titus M., Oh P.J., Gill I.S., Liu Y. (2018). Utilizing Machine Learning and Automated Performance Metrics to Evaluate Robot-Assisted Radical Prostatectomy Performance and Predict Outcomes. J. Endourol..

[15] Bogomolova K., van Merriënboer J.J.G., Sluimers J.E., Donkers J., Wiggers T., Hovius S.E.R., van der Hage J.A. (2021). The effect of a three-dimensional instructional video on performance of a spatially complex procedure in surgical residents in relation to their visual-spatial abilities. Am. J. Surg..

